# Videoconferencing psychotherapy from a psychodynamic point of view. A qualitative analysis

**DOI:** 10.3389/fpsyt.2023.1235478

**Published:** 2023-09-15

**Authors:** Clarissa Laczkovics, Victor Blüml, Nestor Kapusta, Doris Hoffmann-Lamplmair, Erica Casini, Maria Bazan, Miguel Angel Gonzalez Torres, Judit Lendvay, Lina Normandin, Henryk Nowacki, Vladimir Snigur, Stephan Doering, Frank Yeomans, John Clarkin, Emanuele Preti

**Affiliations:** ^1^Department of Child and Adolescent Psychiatry, Medical University of Vienna, Vienna, Austria; ^2^Department of Psychoanalysis and Psychotherapy, Medical University of Vienna, Vienna, Austria; ^3^Comprehensive Center for Clinical Neurosciences and Mental Health, Medical University of Vienna, Vienna, Austria; ^4^Department of Psychology, University of Milano-Bicocca, Milano, Italy; ^5^Polish Society for Psychodynamic Psychotherapy, Cracow Psychodynamic Center, Kraków, Poland; ^6^Department of Neuroscience. University of the Basque Country, San Sebastian, Spain; ^7^Department of Psychiatry, Basurto University Hospital, Bilbao, Spain; ^8^CIBERSAM, Spanish Network of Research in Mental Health, Bilbao, Spain; ^9^Biocruces-Bizkaia Research Institute, Barakaldo, Spain; ^10^Personality Disorders Institute, Weill Cornell Medical College, White Plains, NY, United States; ^11^Columbia Center for Psychoanalytic Training and Research, New York, NY, United States; ^12^École de Psychologie, Université Laval (Québec), Québec, QC, Canada; ^13^Russophone Society for the Transference Focused Psychotherapy, Moscow, Russia; ^14^Department of Psychiatry, Columbia University Vagelos College of Physicians and Surgeons, New York, NY, United States

**Keywords:** pandemic (COVID-19), videoconferencing psychotherapy, psychodynamic psychotherapy, personality disorder, setting (adjustments)

## Abstract

There is a growing interest in delivering videoconferencing psychotherapy (VCP) due to the enormous impact of the COVID-19 pandemic on our lives since the beginning of severe restrictions worldwide in March 2020. Scientific literature has provided interesting results about the transition to remote sessions and its implications, considering different psychotherapy orientations. Less is known about whether and how VCP affects psychodynamic psychotherapeutic approaches and reports on remote work with severe and complex mental health problems such as severe personality disorders are still scarce. The aim of the study was to examine the experiences of psychodynamic psychotherapists, mainly delivering Transference-Focused Psychotherapy (TFP), with the transition and delivery of VCP during the first wave of the COVID-19 pandemic. Four hundred seventy-nine licensed psychotherapists completed an online survey during the peak of the pandemic. Survey data were analyzed using qualitative analysis. Results are presented and discussed concerning advantages and disadvantages regarding the access to psychotherapy, the specificity of the online video setting, bodily aspects, the quality of the therapeutic relationship, the therapeutic process including technical aspects and therapist’s experience. Furthermore, we analyzed and discussed the statements concerning transference and countertransference reactions differentiating between high-level borderline and neurotic patients and low-level borderline patients. Our results support the importance to identify patients who potentially benefit from VCP. Further research including more prospective randomized controlled trials are needed to investigate the therapeutic implications of the findings.

## 1. Introduction

There is a growing interest in delivering psychotherapy remotely via online videoconferencing tools due to the impact of the COVID-19 pandemic and the associated restrictions worldwide on our lives. Prior to the pandemic and the forced necessity for remote contact, the use of videoconferencing psychotherapy (VCP) was not widespread and its perception was controversial, possibly due to limited experience, even though pioneering research groups had shown its efficacy (1–5).

Preliminary studies in the light of the pandemic regarding setting changes report high acceptability of VCP. Therapists and patients did not anticipate how easy the shift from face-to-face psychotherapy to VCP might be and that certain issues might even catalyze positive outcomes (6). The worldwide viral threat and the relief of having a possibility to offer continuous and safe contact might have augmented this optimistic view. Still, psychotherapists are also challenged by working remotely, experiencing feelings of fatigue and isolation, which led to changes in psychotherapeutic technique, such as the management of longer periods of silence (7–11).

VCP has already gained an established evidence in the delivery of cognitive behavioral therapies for posttraumatic stress disorder (2), depression (1), anxiety, and eating disorders (12). However, research on other forms of online psychotherapy such as psychodynamic psychotherapy and on the work with severe and complex mental health problems such as severe personality disorders is still scarce (13). In a face to face setting, patients with severe personality disorders or borderline personality organization have been efficaciously treated with Transference Focused Psychotherapy (TFP) (14–16), which is based on an adapted psychoanalytic model of borderline personality disorder (14, 17–20) performed in a setting twice weekly. Transference refers to the activation of an object relation dyad that exists in the mind of an individual in the course of an interaction with another person. An object relations dyad consists of an internal mental representation of the self in relation to another person and the affect that is associated with those representations. The activation of these internal mental dyads in relation to the therapist is an avenue into understanding the internal world and psychological functioning of the patient. Countertransference refers to emotional reactions in the therapist that are evoked in response to the patient’s interactions with the therapist. The dominant affect that determines the focus of the therapist’s interpretation may be expressed in the patient’s narrative, but also in the patient’s nonverbal behavior or in the therapist’s countertransference. The therapist therefore pays simultaneous attention to the verbal communication, to the nonverbal behavior and to the countertransference, confronting the patient tactfully with contradictions in the communication, especially between verbal and nonverbal communication or between the patient’s communication and the evoked countertransference (21). Therefore, as nonverbal aspects of behavior are extremely important, performing psychodynamic VCP might be especially challenging.

Research on psychodynamic VCP prior to the pandemic was mainly based on single case studies (22–24). From these, authors suggest that patients need to be both externally motivated and capable of making use of remote psychoanalytic treatment. Experiences show that limitations in remote treatment often emerge in patients with substance use disorders and borderline personality symptoms including patients with suicidal tendencies. Psychodynamic VCP requires patients to be capable to maintain the alliance and share the responsibility for the management of the setting (24). On the other hand, advantages were observed for patients who move due to work reasons, or those who live in areas with otherwise limited access to psychoanalytic treatment, or who are hindered by other objective reasons to attend treatment in person.

Ehrlich (22) addresses the importance of establishing a holding frame in VCP (psychoanalysis), as usual in personal encounters, and of addressing the possible denial of loss of part of the experience, which is created by the fact that remote therapy deprives patients of the shared bodily presence with the psychoanalyst. Disadvantages of VCP were seen in the absence of two bodies together, leading to an unbalanced communication in favor of a more verbal cognitive mode. This leads to an additional challenge for psychotherapists compared to face to face sessions, increasing attention difficulties and the experience of inauthenticity and strangeness (23). Also, sustaining conversational silences seemed to be comparatively more difficult in VCP sessions. Ehrlich concludes that videoconference psychoanalysis would produce a sense of forced ego integration, therefore interfering with the access to states of unintegration and its gradual reconciliation by interpretation as part of the psychoanalytic process (22).

In line with these considerations, Jesser et al. published a mixed-methods study on the therapist’s experiences with psychodynamic VCP (8). The authors concluded that psychodynamic VCP has benefits as well as challenges when it comes to establishing and maintaining a stable relationship remotely. They emphasized the impact of the remote setting on psychotherapy, as it directly affects the developing analytic process that involves complex issues such as the management of closeness and distance, presence and absence as well as sensory perceptions.

The aim of the current study was to examine the experiences of psychodynamic psychotherapists, mainly delivering Transference-Focused Psychotherapy (TFP), with the transition and delivery of VCP during the first wave of the COVID-19 pandemic. We aim to investigate the implications of COVID-19 related anxieties as well as the implications of the altered setting on psychotherapeutic treatment. Based on a mixed-methods methodology, we examine here the qualitative reports on obstacles, challenges, and advantages and more specifically changes occurring in psychotherapeutic technique. The quantitative measures are reported separately (25).

A further differentiation between patients with a lower borderline personality organization and patients with a higher borderline or neurotic personality organization, allows to understand specific effects of the changes in setting on the transference and countertransference reactions in both groups and allows examination of patients’ capability to engage in psychodynamic psychotherapy in the context of personality organization.

## 2. Materials and methods

### 2.1. Participants and procedures

We conducted an online survey set up with Qualtrix (Qualtrix LLC). The survey was distributed to all members of the International Society of Transference-Focused Psychotherapy (ISTFP) through the society’s listserv. Additionally, members were asked to forward the survey to colleagues including those using other psychotherapeutic approaches.

Therapists participated on a voluntary basis, after reading and accepting written consent forms. Data were collected between April 25th and June 15th 2020. The study was approved by the IRB of the University of Milan-Bicocca.

### 2.2. Measures

The survey consisted of multiple-choice and 5-point Likert-scale questions as well as several open-ended questions intended for quantitative and qualitative analyses, respectively. It contains questions on sociodemographic aspects, treatment modalities offered pre- and post- COVID-19 (populations treated, usual setting including frequency of sessions, number of patients treated, participation in supervision, etc.), questions pertaining to the experiences of online treatment during the pandemic (advantages and disadvantages, experienced change), and questions on experienced differences in the therapeutic relationship treating patients with severe personality disorder compared to patients with milder forms of personality disturbances. The quantitative data revealed no differences in the responses between TFP and non-TFP psychotherapists, therefore we did not differentiate between these groups in the qualitative analysis.

In this study, only the qualitative results of the following open-ended questions will be presented [see (25) for quantitative results; complete questionnaire available on request]. Sample questions: “Please, describe the advantages you see in delivering online therapy over in-person therapy:,” “Please, describe the disadvantages you see in delivering online therapy over in-person therapy:.” Separately for patients with a high and low level of personality organization/functioning: “I experienced changes in my countertransference – Please, describe:,” “I experienced changes in the patients’ transferential patterns – Please, describe:”

The survey was initially compiled in English and translated into 7 languages (German, Spanish, Italian, French, Hungarian, Polish, Russian). The completion of the questionnaire took approx. 30 min.

### 2.3. Data analysis

The number of responses to the questions ranged from 147 to 368 varying in length between single words (e.g., “none” regarding advantages of online therapy) to multiple paragraphs.

The response data were analyzed using qualitative content analysis (26) by two researchers (VB and NDK) separately with experience in conducting qualitative research as well as being psychiatrists and trained psychoanalysts. Both researchers involved share a modern object relations psychoanalytic orientation and have taken part in regular clinical and scientific meetings at the Department of Psychoanalysis and Psychotherapy of the Medical University in Vienna in which theoretical and clinical aspects of online treatment modalities and the impact of the pandemic on the psychotherapeutic process were discussed from a psychoanalytic viewpoint. This theoretical background created a vertex of understanding during the analysis of the qualitative data.

The researchers first familiarized themselves separately with the data by reading all responses and discussing relevant aspects repeatedly. Then, preliminary category definitions and coding rules were formulated inductively in joint work. Next, the first 100 responses to each question were analyzed separately by both researchers by deductively subsuming them under the established categories. In a next step, the coding was discussed, category definitions and coding rules with low agreement were refined and new categories were formulated when necessary. Subsequently, the remaining responses were rated by each single rater using the established categories and coding rules iteratively discussing the progress. If the material made the addition of new categories necessary or a response was difficult to allocate to the established categories, this was discussed until agreement could be reached. Especially poignant answers were marked, and frequency of response categories were counted to represent the agreement among the survey participants. In a final step, the categories were grouped into overarching themes on a higher level of abstraction taking preexisting research into account [*cf.* especially (8), but also (27)] allowing for better comparison of the data: access to psychotherapy, specificity of the online video setting, bodily aspects, the quality of the therapeutic relationship, the therapeutic process and the therapist’s experience. Frequency of responses in each category was counted; only those specific responses that had a frequency greater than five were listed in the resulting Tables 1–3.

**Table 1:** Advantages and disadvantages of VCP.

Advantages (*n* = 326)	Frequency	Disadvantages (*n* = 368)	Frequency
None	21	None	0
*Access to psychotherapy*	*239*		*78*
Convenience	119	Technical issues, Data protection	58
Continuity	76	No transitional period (attunement, fading out)	14
Safety/less infection risk	29		
Easier to keep timeframe/schedule	11		
*Specificity of the online video setting*	*36*		*92*
Insight into personal environment/privacy	24	Privacy issues (private room)	37
Video advantages (e.g., details of face mimics)	9	Loss of total perspective	25
		Loss of safety and protection	20
*Bodily aspects*	*0*		*216*
		Loss of nonverbal channels of communication	162
		Loss of corporeality	44
		Loss of eye contact	9
*Therapeutic relationship*	*83*		*96*
More distance/less contact	17	Loss of emotional contact/ more distant	40
Less anxiety	14	Less containment	16
More focused/concentrated	10	More distance	11
More proximity	6	Transference less intense, superficial	15
Less shame	5	Lack of immediacy	6
More introspective and reflective	5	Difficulty with management of suicidality/ psychosis difficult	5
Transference: less intense	5	Less engagement	5
*Therapeutic process/technical aspects*	*69*		*49*
Facilitation of opening up	38	Intolerance of silence	17
More freedom to think	9	Transference interpretations/confrontations more difficult	8
New material	7	More acting out	7
More focus on verbal material	5	More cognitive/psychoeducative	7
*Therapist’s experience/Countertransference*	*28*		*115*
More focused/ concentrated	10	More exhausting	40
Easier to keep timeframe/ schedule	9	More distractions (patient and therapist)	22
Easier to apply	5	Restricted CT	11
		Difficulties recognizing emotions	10
		More difficult maintaining the frame	7
		More effort needed to stay connected	7
		More self-control/concentration	7

VCP, videoconferencing psychotherapy.

**Table 2:** Changes of transference and countertransference reactions transitioning high level borderline and neurotic patients from face to face to VCP.

Transference (*T*, *n* = 147)	Frequency	Countertransference (CT, *n* = 156)	Frequency
None/little	26	None/little	18
Increased positive *T*		Increased positive CT	
More free association/ more open	6	More positive attitude/empathy/more valued/motivated	6
More gratitude β	5		
More positive/friendly	5		
*Increased negative T*		*Increased negative CT*	
More paranoid/distrust/devaluation	8	More negative feelings: anger/dysphoria/annoyance/reluctancy	11
More anger/tension	6	More irritated/less calm	6
*Dependency/attachment*		*Distance*	
More needy/vulnerable/demanding/helplessness	23	Less emotional/ less affect/ less intense	12
More idealization	7	Less contact	7
*Emotional distance*		*Therapeutic attitude/technique*	
Less intimacy/less connected	10	More supportive	32
Less intense	9	More concerned	11
More intense/more emotional intimacy	7	Less confrontational	5
More superficial	6	More psychoeducative/less neutral	5
*Pandemic*		*Pandemic*	
Worries/concern about health of therapist	12	Shared reality/solidarity/horizontal relationship	5
Shared reality/sense of solidarity	5		
*Setting*		*Setting*	
Curiosity about private life	4	Occupied/irritated with technical issues	4
		More exhaustion/ less concentrated	4

VCP, videoconferencing psychotherapy.

**Table 3:** Changes of transference and countertransference reactions transitioning low level borderline patients from face to face to VCP.

Transference (*T*, *n* = 203)	Frequency	Countertransference (CT, *n* = 239)	Frequency
None	10	Little/none	11
*Increased positive T*		*Increased positive CT*	
More positive feelings, less aggression	17	More caring / compassion	5
More free association/ more open	12	Calmer/ more patient	5
Less paranoia	8		
More closeness	5		
Less acting out	5		
Less anxiety	5		
*Increased negative T*		*Increased negative CT*	
More aggression	20	More helplessness/resignation	19
(Fear of) dropout	15	More irritated; less calm	12
More resentment	15	More anxiety	12
More paranoia	15	Lower frustration tolerance/ more impatient	7
More boundary enactments/risk behavior	12	More anger	7
More anxiety	6	More annoyed	6
More depressed	6	*Distance*	
More negative transference	6	More distant	26
More controlling	5	More difficult to recognize CT	13
*Dependency/attachment*		Less interest/more boredom	9
More needy/clinging	21	More superficial/less vital	8
Fear of abandonment/ attachment insecurity	14	Increased intensity / more intimate	6
More regression	11	More casual	5
More idealization (analyst as savior)	10	*Therapeutic attitude/technique*	
*Emotional distance*		More supportive	27
More superficial/less intensity	27	More concerned	12
More casual	7	Harder to maintain therapeutic neutrality	8
Less engagement	6	Difficulty in maintaining setting	5
More withdrawn	5		
*Pandemic*		*Pandemic*	
Shared experience/horizontal relationship	6	Shared experience/ horizontal relationship	7
Concern about the object	5	*Setting*	
*Setting*		More exhausting	6
More curiosity about private life of therapist	8	Frustration/irritation with technical issues	5

VCP, videoconferencing psychotherapy.

## 3. Results

### 3.1. Participants

Altogether, 479 therapists from 25 countries completed the survey (mean age: 49.46 years, *SD* = 12.42, range 24–91) out of which 67.2% (*n* = 322) were females and 32.6% (*n* = 156) were males, with one participant self-identifying as “other.” 61.8% (*n* = 296) were TFP-therapists and 38.2% (*n* = 183) practiced another form of psychotherapy. The study participants consist of highly experienced therapists with a self-reported mean of 14.38 years after certification (SD = 11.68, range 0–60).

The results of the qualitative data are shortly presented in this section and extensively reported and discussed in the discussion section, as results and discussion are closely linked in order to avoid redundancies.

### 3.2. Advantages and disadvantages

Advantages and disadvantages are listed according to specific categories (see Table 1).

Access to psychotherapy was seen in the majority as positive (239 vs. 78). The specificity of the remote setting was rated by 92 as a disadvantage vs. 36 who rated advantages. Bodily aspects regarding VCP were experienced exclusively as a disadvantage (216 vs. 0). The quality of the therapeutic relationship was rated as beneficial by 83 and as deteriorating by 96. Regarding the therapeutic process including technical aspects, 69 saw advantages in delivering VCP vs. 49 who stated disadvantages. Regarding therapists’ experience including countertransference, most psychotherapists emphasized the disadvantages (115 vs. 28). Specific ratings related to these categories are listed in Table 1.

### 3.3. Transference and countertransference reactions

Ratings to the specific changes in transference and countertransference reactions when shifting from face-to-face setting to VCP with higher borderline or neurotic patients are shown in Table 2. Results for low level borderline patients are shown in Table 3.

#### 3.3.1. Transference and countertransference in patients with a higher personality structure, either neurotic, or high borderline level

Changes in transference and countertransference reactions were seen by most of the psychotherapists, though still some perceived no changes due to the setting switch.

Patients were perceived as more in need and dependent with a higher tendency to idealize the therapist as a “savior.” Some therapists found their patients to be more positive and grateful, opening up to free association, some perceived increased paranoid reactions, devaluation and increased anger. Many described the contact less intimate, less intense, distant and superficial. Patients worried about health issues of the therapist sharing the same experience, leading to more curiosity about the therapists’ private life, to more casual encounters, describing overall a more horizontal relationship. Therapists were seen more as real people.

Changes in countertransference were rated as little by a large proportion of therapists. Countertransference reactions ranged from being more supportive, less confrontational, more intellectualized, less emotional and more focused on external reality to being more anxious and concerned. Some therapists felt a closer connection with their patients, but the majority felt less connected. Negative feelings being perceived were anger and exhaustion.

#### 3.3.2. Transference and countertransference in patients with a lower borderline personality structure

Few therapists perceived no or little changes in transference and countertransference reactions. Some therapists perceived an increase of positive transference reactions, patients being grateful and friendly, opening up with free association. However, a majority describe strong negative emotions as highly dominant ranging from anger and devaluation to paranoid feelings. There was an increase in anxious feelings with an intensified fear of abandonment and loss. Furthermore, an increase in the use of primitive defense mechanisms like splitting and denial was observed, as well as an increase of depersonalization, derealization and dissociative behaviors. Boundary enactments, risky behavior and fear of drop out on the one hand and neediness, regression and idealization on the other hand challenged the setting. Many therapists saw the patients being more distant, superficial and less engaged in the process. Others perceived a more horizontal encounter and more curiosity about the therapist’s private life.

Countertransference reactions included feelings of anxiety and helplessness. The vast majority of therapists reported they felt less in contact and equally distant and superficial. Negative reactions such as anger and annoyance were observed by many therapists, but these feelings did not lead to more confrontative behavior, but rather to being more supportive and focused on external reality. It was felt to be harder to stick to technical neutrality, some described it being harder to end sessions. Therapists perceived themselves overall as more cognitive, with lower frustration tolerance and fear of patients to drop out. Sessions were experienced as exhausting, therapists being more concerned in the relationship.

## 4. Discussion

### 4.1. Advantages and disadvantages

#### 4.1.1. Remote access to psychotherapy

Remote access to psychotherapy was seen on the most part of surveyed therapists to be convenient. Access was judged to be easier and safer regarding infection risk. An advantage was seen in the flexibility, which might have led to less missed sessions and more punctuality. On the other hand, therapists were concerned about technical issues and safety questions regarding data protection and confidentiality. These results are in line with most research, especially regarding studies performed at the beginning of the pandemic, when videoconferencing was the only safe way to enable the maintenance of an ongoing psychotherapy (7–11, 28–32).

#### 4.1.2. Specificity of the videoconferencing setting

In analytic terms, setting is the essential background which provides the necessary containment and stimulus for the unfolding of the patient’s transference (33). In face-to-face settings, the therapist sets it, maintains it, and has the primary responsibility for it. The specificity of the videoconferencing setting is that the therapist cannot control the environment and therefore ensure safety in which the patient receives the therapy (34).

The office space is lacking, as therapy takes place in the private home of patients, and often also of psychotherapists. The insight into the private environment was rated as an advantage by some therapists, gaining more personal information. This information could be therapeutically used by understanding it as an implicit expression (camera arrangements, clothing, items unveiled, etc.). On the other hand, most therapists refer to the disadvantages of such a setting, namely that it was often hard for the patients to establish privacy, and the loss of the familiar setting in the office was mentioned by many therapists. With it, therapists describe a loss of control over the therapy environment and setting, therefore conduct rules had to be re-negotiated. Jesser et al. (8) refer also to the loss of the transitional space in the office, where patients could symbolically leave stressful content behind.

#### 4.1.3. Bodily aspects

When therapy is performed in a shared physical space, the patient and therapist can make use of implicit communication. The non-verbal behavior and especially the discrepancies of verbal and non-verbal channels are ways of expressing the unconscious conflictual situation. Especially patients with more severe structural personality disturbances, who have difficulties in establishing and maintaining a stable differentiation of the self from the object, as well as difficulties in symbolization, are understood to ‘project’ inner tensions powerfully into the therapists’ body leading to somatic countertransference reactions (33, 35). The understanding of these dynamics is usually an important aspect supporting the development of the capacity to symbolize (36).

As expected, the vast majority of the respondents refer to the loss of nonverbal channels of communication as the major disadvantage of VCP. They describe the loss of the shared therapeutic space as an embodied experience of physical presence as a loss of the whole perspective (22, 31, 32, 37, 38). The atmosphere, the smell, eye contact, the impossibility of handing a tissue made access to affects more difficult (23). However, a few therapists do not see the screen as an obstacle, but in opposite as an advantage, helping to stay more focused on the expressed verbal material. Some even describe more proximity behind the screen, experiencing the screen as a protective boundary. Suler created the term online disinhibition effect (39), and explored six factors that lead to disinhibited interaction online. Two of those factors might also be relevant for online psychotherapy, dissociative imagination (“the other is not real”) and minimization of authority (“I can act freely”) (34).

The following statements illustrate the above interpretation:

*“Limitation of nonverbal stimuli is a big disadvantage. And uncertainty of available nonverbal stimuli (if my patient had tears in their eyes while in my office then I was sure about it, when we are online and I think there are tears and they do not feel it, because of dissociation, then I cannot comment on that, I have to rely on patient’s agreeing or disagreeing). There’s no body, and because of that some impulses are seen as less threatening (both libidinal and aggressive) – so patients are more “free” in some ways. At first it makes an interesting impression (but can also encourage acting out) but in general I believe that it limits our ability to touch and work through patient’s anxiety.”*

*“On the one hand, the screen serves as a protection (for instance, from sexual and aggressive actions) but on the other hand it may become an obstacle to establishing trust and closeness (probably not always or to various degrees for various patients). Some of the controlling and protective functions are delegated to the screen. There seems to be something like a protected contract […].”*

#### 4.1.4. Therapeutic relationship

The advantage of providing continuity was stressed as an important issue arising from the opportunity of VCP. Similarly, a safe contact, in contrast to being at risk of contagion, was appreciated. Some respondents saw patients opening up and found emotional expression to be facilitated by VCP, which might again refer to an online disinhibition effect (39), but could also refer to a more cognitive mode of communication. In line with the latter explanation, the relationship was found to be less defensive, less anxious and paranoid and therapists experienced a better alliance. The majority of the therapists reported a loss of emotional contact and felt more at distance, which might at first sight seem to be opposing to the experience of facilitated emotional expression but actually might be in line with a switch to a more cognitive approach arising in some patients as a consequence of VCP. Difficult situations such as suicidality, acute crisis and psychotic decompensation were harder to be managed and contained. As pointed out previously (24), this might refer to the need of patients to engage in the therapeutic process and the ability to care for a safe setting, which might have been a difficult task for lower level borderline patients with chronic suicidal ideation, as known from clinical experience.

The following statements refer to those difficulties:

A therapist describes a *“difficulty in containing emotions during the session, in enduring silences, in containing the appearance of transferential affective movements which are more anxiety-provoking for patient and teletherapy therapists than in person sessions.”*

*“With patients on the borderline and/or complex trauma spectrums, my physical absence is experienced as an abandonment. Most if not all sessions have been addressing the disruption and anger of feeling me far away and fears I will never return (likely a paranoid infantile state regression).”*

#### 4.1.5. Therapeutic process

TFP-treatment focusses on the integration of split-off self and object representations. The therapist clarifies and interprets from a position of technical neutrality utilizing countertransference as a major therapeutic tool (21). The lesser the capacity to symbolize and verbalize a patient has due to structural deficits, the more important it is to focus on non-verbal communication including somatic reactions, body and facial expressions.

Some therapists were concerned about the therapeutic process, as there was no transitional period of attunement and fading out before and after the session. They also found a new intolerance for silence and reactively less silence overall. The process was mostly experienced as being more supportive, more cognitive and intellectual. On the side of the patient, therapists observed more acting out outside the sessions and inhibitions in free association. This is in line with previous research describing a deeper process being obstructed in an online setting (8). Furthermore, the distancing effect of VCP allows some patients to hide perceived feelings of helplessness and neediness more efficiently (6). In other cases, therapists see an advantage in less intense transference reactions, leaving more freedom to think and to a form of disinhibition with more free association revealing new material (6).

Therapists describe difficulties in emotion recognition and having to put more effort in being connected. The process is described as restricted and distracted, more psycho-educative and more factual. Regression is seen as restricted. Most therapists found themselves to be exhausted, few found it easier to stay focused, easier to work and were more relaxed.

The following quotes illustrate these thoughts:

A therapist describes how “*some patients find it difficult to read me which makes them very anxious. Somehow it is more difficult to listen to my ‘gut feeling’. Projective identification is more difficult to feel.”*

*“I, as a psychologist, feel exhausted and less attuned to my patients’ nonverbal body cues and so prefer in-person sessions. Although not nearly the same as this, online therapy has the feel of a mother parenting through a screen: what is said may be heard and considered, but it feels as though it is not as deeply known and integrated. I am seeing literally parts of my patients rather than the whole person made evident by what is absent and hidden out of the camera.”*

#### 4.1.6. Therapists experience

Overall the therapists experienced psychodynamic VCP as more exhausting and -in many ways- restricted. Some found themselves to be more concentrated and focused, though this was also seen as a disadvantage. They found their countertransference reactions to be restricted and more effort was needed to stay in contact with their patients and to recognize emotions.

On a meta-level we found that many comments by therapists contained narratives of loss experiences. As mentioned above, loss of nonverbal channels of communication and with it the loss of the embodied experience of the total perspective, the loss of the safe personal encounter, being emotionally in contact, as well as the loss of control over the therapeutic setting was observed. Mourning is a central part in psychodynamic psychotherapies, usually within a holding environment with physical presence. The working through of these losses in the therapeutic process is seen to be highly relevant when initiating or changing the setting into VCP (22, 34). Since the majority of therapists included in the current study worked with patients with personality disorders, it might be especially challenging to address these issues.

### 4.2. Therapists’ and patients’ reactions

#### 4.2.1. Transference and countertransference in patients with a higher personality structure, either neurotic, or high borderline level

In high level borderline and neurotic patients, the majority of therapists judge the remote therapeutic relationship to be less intimate and emotional, though with overall less changes in positive or negative transference and countertransference reactions. The shared experience of lockdown situations and adapting to the danger of the pandemic makes working with these patients more supportive and less confrontational, working on realistic anxieties and being more rational and intellectualizing. These patients are occupied with their own helplessness and vulnerability as well as with concerns and worries about the health of the therapist. This might indicate that perceived changes are not mainly caused by the setting change but also by the pandemic situation itself, therapist and patient sharing the same reality. Still, the question of emotional superficiality and increased cognitive work going along with VCP has been debated also before the pandemic (22–24).

#### 4.2.2. Transference and countertransference in patients with a lower borderline personality structure

With lower level borderline patients, the experience is markedly different.

Overall, transference reactions to VCP were judged as predominantly negative, including more aggression, more resentment, paranoia, and boundary enactments.

This led to increased negative countertransference reactions that could not be addressed and interpreted. Therapeutic attitude changed similarly as in therapies of high-level borderline patients, being more supportive and less confrontational leading to a loss of emotional closeness.

These experiences reflect the challenge to maintain a holding and containing setting, where it is felt to be safe, which is a precondition to confront and to interpret transference dynamics, especially for patients with severe structural deficits. Prior to the pandemic, contraindication for VCP was seen for patients suffering from personality disorders, severe depression with suicidality and for those with unstable interpersonal relationships (22, 40). Our results equally point to the need to differentiate between those patients who might profit from VCP from those that urgently need the holding frame of a face to face setting.

### 4.3. Limitations

It has to be taken into account that the experiences with newly established VCP are intertwined with the experience of the anxiety-laden situation of the COVID-19 pandemic, being anxious about one’s own health and the health of significant others, as well as having to face stressful changes in forced isolation. The opportunity to maintain a stable working relationship with patients via videoconferencing might therefore have led to increased acceptance of VCP. Additionally, circumstances meant a unique united perspective, as psychotherapist and patient shared the same fearful external reality leading to a more horizontal encounter that might counteract therapeutic regression and could have led to the experience of less intense, less intimate relationships which appeared to be more supportive and intellectualized. This has to be kept in mind when interpreting the results, which reflect not only reactions to the VCP setting, but also reactions to the threatening pandemic situation and the forced character of the setting shift. Therefor results cannot be generalized and further research after the pandemic is needed.

## 5. Conclusion

Videoconferencing has many advantages regarding the access to psychotherapy. The therapeutic setting, usually set and maintained in the office by the therapist, shifts more toward shared responsibility of both patient and the therapist. The loss of the shared bodily presence seems to be a disadvantage especially for psychodynamic psychotherapies, as the intensified focus on verbal communication leads to a more cognitive approach. Our results show that particularly patients with lower-level borderline personality structure react to the change of the therapeutic setting with stronger feelings of insecurity and helplessness, with more aggressive and paranoid transference reactions in the course. Additionally, these patients are thought to be especially affected by pandemics and this might lead to worse outcome (41). Therapists stated enormous obstacles in containing these strong feelings and maintaining their therapeutic capacities due to the pressure to maintain therapeutic relationships under massive societal changes. Our results support and document the perception that videoconferencing has an impact on various aspects of the therapeutic setting, technique and the way patients cope with such changes. Since the COVID-19 pandemic, several guidelines for the delivery of VCP have been published, including recommendations to screen patients for eligibility for this form of treatment (42–44). The current study underscores the importance to clarify which patients are not harmfully affected by VCP and those who may unproblematically continue therapy by means of modern digital advancements. Additional research including prospective randomized controlled trials are needed to investigate these open questions.

## Data availability statement

The raw data supporting the conclusions of this article will be made available by the authors, without undue reservation.

## Ethics statement

The studies involving humans were approved by IRB of the University of Milan-Bicocca. The studies were conducted in accordance with the local legislation and institutional requirements. The participants provided their written informed consent to participate in this study.

## Author contributions

FY, JC, SD, and EP: conception and design of the study. CL, VB, NK, DH-L, EC, MB, MT, JL, LN, HN, VS, and SD: acquisition and analysis of data. CL and VB: drafting the manuscript or tables. All authors have reviewed and approved the final manuscript.

## References

[1] Berryhill MB Culmer N Williams N Halli-Tierney A Betancourt A Roberts H. Videoconferencing psychotherapy and depression: a systematic review. Telemed J E Health. (2019) 25:435–6. doi: 10.1089/tmj.2018.0058, PMID: 30048211

[2] Howard R Berry K Haddock G. Therapeutic alliance in psychological therapy for posttraumatic stress disorder: a systematic review and meta-analysis. Clin Psychol Psychother. (2022) 29:373–9. doi: 10.1002/cpp.2642 34237173

[3] Norwood C Moghaddam NG Malins S Sabin-Farrell R. Working alliance and outcome effectiveness in videoconferencing psychotherapy: a systematic review and noninferiority meta-analysis. Clin Psychol Psychother. (2018) 25:797–8. doi: 10.1002/cpp.2315, PMID: 30014606

[4] Simpson SG Reid CL. Therapeutic alliance in videoconferencing psychotherapy: a review. Aust J Rural Health. (2014) 22:280–9. doi: 10.1111/ajr.12149, PMID: 25495622

[5] Tonn P Reuter SC Kuchler I Reinke B Hinkelmann L Stockigt S. Development of a questionnaire to measure the attitudes of laypeople, physicians, and psychotherapists toward telemedicine in mental health. JMIR Ment Health. (2017) 4:e39. doi: 10.2196/mental.6802, PMID: 28974485 PMC5645642

[6] Merchant J. Working online due to the COVID-19 pandemic: a research and literature review. J Anal Psychol. (2021) 66:484–5. doi: 10.1111/1468-5922.12683, PMID: 34231903 PMC8441839

[7] Bock MM Graf T Woeber V Kothgassner OD Buerger A Plener PL. Radical acceptance of reality: putting DBT(R)-a skill groups online during the COVID-19 pandemic: a qualitative study. Front Psych. (2022) 13:617941. doi: 10.3389/fpsyt.2022.617941, PMID: 35546945 PMC9082632

[8] Jesser A Muckenhuber J Lunglmayr B. Psychodynamic Therapist’s subjective experiences with remote psychotherapy during the COVID-19-pandemic-a qualitative study with therapists practicing guided affective imagery, hypnosis and autogenous relaxation. Front Psychol. (2021) 12:777102. doi: 10.3389/fpsyg.2021.777102 35069358 PMC8777098

[9] McBeath AG du Plock S Bager-Charleson S. The challenges and experiences of psychotherapists working remotely during the coronavirus pandemic. Couns Psychother Res. (2020) 20:394–5. doi: 10.1002/capr.12326, PMID: 32837329 PMC7361364

[10] Mitchell EJ. Much more than second best: therapists’ experiences of videoconferencing psychotherapy. Euro J Qual Res Psychother. (2020) 10:121–5.

[11] Simpson S Richardson L Pietrabissa G Castelnuovo G Reid C. Videotherapy and therapeutic alliance in the age of COVID-19. Clin Psychol Psychother. (2021) 28:409–1. doi: 10.1002/cpp.2521, PMID: 33037682 PMC7675483

[12] Thomas N McDonald C de Boer K Brand RM Nedeljkovic M Seabrook L. Review of the current empirical literature on using videoconferencing to deliver individual psychotherapies to adults with mental health problems. Psychol Psychother. (2021) 94:854–3. doi: 10.1111/papt.12332, PMID: 33620133 PMC8451850

[13] Broadbear JH Heidari P Dharwadkar NP Cheney L Rao S. Telehealth psychotherapy for severe personality disorder during COVID-19: experience of Australian clinicians. Global J Health Sci. (2021) 13:61. doi: 10.5539/gjhs.v13n12p61

[14] Clarkin JF Levy KN. Psychotherapy for patients with borderline personality disorder: focusing on the mechanisms of change. J Clin Psychol. (2006) 62:405–10. doi: 10.1002/jclp.20238, PMID: 16470611

[15] Doering S Horz S Rentrop M Fischer-Kern M Schuster P Benecke C. Transference-focused psychotherapy v. treatment by community psychotherapists for borderline personality disorder: randomised controlled trial. Br J Psychiatry. (2010) 196:389–5. doi: 10.1192/bjp.bp.109.070177, PMID: 20435966

[16] Linehan MM. Cognitive-behavioral treatment of borderline personality disorder. New York: Guilford Press (1993).

[17] Clarkin JF Levy KN Lenzenweger MF Kernberg OF. Evaluating three treatments for borderline personality disorder: a multiwave study. Am J Psychiatry. (2006) 164:922–8. doi: 10.1176/ajp.2007.164.6.922, PMID: 17541052

[18] Cristea IA Gentili C Cotet CD Palomba D Barbui C Cuijpers P. Efficacy of psychotherapies for borderline personality disorder: a systematic review and Meta-analysis. JAMA Psychiat. (2017) 74:319–8. doi: 10.1001/jamapsychiatry.2016.4287 28249086

[19] Kernberg OF. Severe personality disorders. New Haven: Yale University Press (1984).

[20] Levy KN Clarkin JF Yeomans FE Scott LN Wasserman RH Kernberg OF. The mechanisms of change in the treatment of borderline personality disorder with transference focused psychotherapy. J Clin Psychol. (2006) 62:481–1. doi: 10.1002/jclp.20239, PMID: 16470612

[21] Kernberg OF Yeomans FE Clarkin JF Levy KN. Transference focused psychotherapy: overview and update. Int J Psychoanal. (2008) 89:601–10. doi: 10.1111/j.1745-8315.2008.00046.x 18558958

[22] Ehrlich LT. Teleanalysis: slippery slope or rich opportunity? J Am Psychoanal Assoc. (2019) 67:249–9. doi: 10.1177/0003065119847170 31088141

[23] Gutierrez L. Silicon in ‘pure gold’? Theoretical contributions and observations on teleanalysis by videoconference. Int J Psychoanal. (2017) 98:1097–20. doi: 10.1111/1745-8315.12612, PMID: 27991668

[24] Scharff JS. Clinical issues in analyses over the telephone and the internet. Int J Psychoanal. (2012) 93:81–95. doi: 10.1111/j.1745-8315.2011.00548.x, PMID: 22320136

[25] Preti E Casini E Bazan M Blüml V Torres MAG Lendvay J. Transition to online transference-focused psychotherapy during the COVID-19 pandemic. Psychoanal Psychol. (2023).

[26] Mayring P. Qualitative content analysis: a step-by-step guide. London: Sage (2021).

[27] Pugh M Bell T Dixon A. Delivering tele-chairwork: a qualitative survey of expert therapists. Psychother Res. (2021) 31:843–8. doi: 10.1080/10503307.2020.1854486 33315529

[28] Bekes V Aafijes-van Doorn K. Psychotherapists’ attitudes toward online therapy during the COVID-19 pandemic. J Psychother Integr. (2020) 30:238–7. doi: 10.1037/int0000214

[29] Bekes V Aafjes-van Doorn K Prout TA Hoffman L. Stretching the analytic frame: analytic Therapists’ experiences with remote therapy during COVID-19. J Am Psychoanal Assoc. (2020) 68:437–6. doi: 10.1177/0003065120939298, PMID: 32589042

[30] Fernandez-Alvarez J Fernandez-Alvarez H. Videoconferencing psychotherapy during the pandemic: exceptional times with enduring effects? Front Psychol. (2021) 12:589536. doi: 10.3389/fpsyg.2021.589536, PMID: 33679513 PMC7933024

[31] Gumz A Kanal S Unser A Kastner D Beck-Hiestermann FML. “achieving closeness in video treatment despite distance” how did psychotherapists experience the use of video treatment in times of COVID-19? Psychotherapeut. (2021) 66:382–7. doi: 10.1007/s00278-021-00529-y, PMID: 34456515 PMC8386344

[32] Schuster R Topooco N Keller A Radvogin E Laireiter A-R. Advantages and disadvantages of online and blended therapy: replication and extension of findings on psychotherapists’ appraisals. Internet Interv. (2020) 21:100326. doi: 10.1016/j.invent.2020.100326, PMID: 32477885 PMC7251770

[33] Lemma A. The body of the analyst and the analytic setting: reflections on the embodied setting and the symbiotic transference. Int J Psychoanal. (2014) 95:225–4. doi: 10.1111/1745-8315.12147, PMID: 24506063

[34] Lemma A. The digital age on the couch: Psychoanalytic practice and new media. London: Routledge (2017).

[35] Hartung T Steinbrecher M. From somatic pain to psychic pain: the body in the psychoanalytic field. Int J Psychoanal. (2018) 99:159–10. doi: 10.1111/1745-8315.12651 28342209

[36] Leuzinger-Bohleber M. Biographical truths and their clinical consequences: understanding ‘embodied memories’ in a third psychoanalysis with a traumatized patient recovered from severe poliomyelitis. Int J Psychoanal. (2008) 89:1165–87. doi: 10.1111/j.1745-8315.2008.00100.x, PMID: 19126084

[37] Bizzari V. Absent bodies: Psychotherapeutic challenges during COVID-19. Psychopathology. (2022) 55:1–10. doi: 10.1159/000524711 35649386 PMC9393794

[38] Wiener J. Reflections on the role of the analytic setting in the light of COVID-19. J Anal Psychol. (2021) 66:793–2. doi: 10.1111/1468-5922.12717, PMID: 34758134 PMC8652550

[39] Suler J. The online disinhibition effect. Cyberpsychol Behav. (2004) 7:321–6. doi: 10.1089/1094931041291295 15257832

[40] Harris E Younggren J. Risk management in the digital world. Prof Psychol Res Pract. (2011) 42:412–8. doi: 10.1037/a0025139

[41] Preti E Di Pierro R Fanti E Madeddu F Calati R. Personality disorders in time of pandemic. Curr Psychiatry Rep. (2020) 22:80. doi: 10.1007/s11920-020-01204-w, PMID: 33170391 PMC7652908

[42] Puspitasari AJ Heredia D Gentry M Sawchuk C Theobald B Moore W. Rapid adoption and implementation of telehealth group psychotherapy during COVID 19: practical strategies and recommendations. Cogn Behav Pract. (2021) 28:492–6. doi: 10.1016/j.cbpra.2021.05.002 34188434 PMC8223010

[43] Sheperis D Smith A. Telehealth best practice: a call for standards of care. J Techn Counsel Educ Super. (2021) 1:27–35.

[44] Van Daele T Karekla M Kassianos AP Compare A Haddouk L Salgado J. Recommendations for policy and practice of Telepsychotherapy and E-mental health in Europe and beyond. J Psychother Integr. (2020) 30:160–3. doi: 10.1037/int0000218

